# Circadian rhythm of markers of bone turnover in patients with chronic kidney disease

**DOI:** 10.1016/j.bonr.2022.101593

**Published:** 2022-05-25

**Authors:** D. Hansen, I. Bressendorff, A. Nordholm, Astrid Sand Møller, T.W. Klausen, N.R. Jørgensen

**Affiliations:** a Department of Nephrology, Copenhagen University Hospital - Herlev and Gentofte, Copenhagen, Denmark; b Institute of Clinical Medicine, University of Copenhagen, Denmark; c Department of Nephrology, Copenhagen University Hospital - Rigshospitalet, Copenhagen, Denmark; d Department of Hematology, Copenhagen University Hospital - Herlev and Gentofte, Copenhagen, Denmark; e Department of Clinical Biochemistry, Copenhagen University Hospital – Rigshospitalet Glostrup, Copenhagen, Denmark

**Keywords:** Biochemical markers of bone turnover, Chronic kidney disease, Renal osteodystrophy, Circadian clock

## Abstract

Patients with chronic kidney disease (CKD) have a high risk of bone fractures. A circadian rhythmicity in turnover and mineralization of bone appears to be of importance for bone health. In CKD disturbances in the circadian rhythm of various functions has been demonstrated and indeed the circadian rhythm in the mineral metabolism is disturbed. The aim of the present study was to compare the circadian rhythm of bone turnover markers in ten patients with CKD to ten healthy controls. Bone turnover markers (C-terminal telopeptide of type I collagen, tartrate-resistant acid phosphatase 5b, N-terminal propeptide of type I procollagen, bone alkaline phosphatase and osteocalcin) were measured every third hour for 24 h. All bone turnover markers displayed a significant circadian rhythm in both groups and there were no significant differences in the rhythmicity between the two groups (no group*time interaction). As expected, due to the reduced renal clearance, the overall level of C-terminal telopeptide of type I collagen and osteocalcin was higher in CKD compared to the healthy controls. The present study suggests that disturbances in the circadian rhythm of bone turnover do not explain the metabolic bone disease and increased risk of fractures in CKD.

## Highlights

• Patients with chronic kidney disease (CKD) have a high risk of bone fracture.
• Disturbances in the circadian rhythm of bone turnover may be of importance.
• We found a circadian rhythm of bone turnover markers in patients with CKD.
• The rhythmicity did not differ between patients with CKD and healthy controls.
• The circadian rhythmicity of bone turnover markers is not disturbed in CKD.

## 1 Introduction

The risk of fractures progressively increases as kidney function declines and in end-stage kidney disease (ESKD) the risk of fractures is 2 to 3 times higher compared to the general population (Hansen et al., 2016; Pimentel et al., 2017). Additionally, the risks of prolonged hospitalization and death after a bone fracture are elevated in patients with chronic kidney disease (CKD) (Tentori et al., 2014). Renal osteodystrophy is an alteration in bone morphology, which occurs in patients with CKD and encompasses a wide spectrum of bone abnormalities present in more than two thirds of patients with ESKD (Keronen et al., 2019; Salam et al., 2018). By the time they reach ESKD, almost all patients have disturbances in mineral metabolism (Hansen, 2012; Isakova et al., 2020; Levin et al., 2007).

Bone mineralization appears to be regulated by a circadian rhythm. A circadian rhythm exists in mineral deposition in calvarial organ cultures, which correlates with clock gene activity (McElderry et al., 2013), and a diurnal expression pattern of clock- and osteoclast-related genes such as *Rankl* and *Opg* has been demonstrated in bones from healthy mice (Schilperoort et al., 2020). Disturbances in the circadian rhythm may also seriously affect bone health. Night shift work is associated with osteoporosis, and in the Nurses' Health Study an increased fracture risk was found in the nurses with rotating shifts of night work (Bukowska-Damska et al., 2019; Feskanich et al., 2009).

Several bone turnover markers have been shown to exhibit circadian rhythmicity, which may reflect the cyclic pattern of intrinsic circadian rhythm and external signals (Greenspan et al., 1997; Pedersen et al., 1995; Qvist et al., 2002; Redmond et al., 2016; van der Spoel et al., 2019; Wichers et al., 1999). In CKD, a disturbed rhythmicity affects plasma 1,25(OH)_2_ vitamin D and urinary calcium excretion in humans (Jacobsen et al., 2021), and parathyroid hormone (PTH), phosphate and fibroblast growth factor-23 in rats (Nordholm et al., 2019). Knowledge regarding a circadian rhythm of bone turnover markers is important as these appear to be better predictors of bone turnover than PTH (Jørgensen et al., 2021) and have been suggested for use in guiding treatment decision for renal osteodystrophy (Evenepoel et al., 2021).

Thus, the aim of the present study was to investigate and compare the circadian rhythm of bone turnover markers in patients with CKD and healthy controls.

## 2 Methods

### 2.1 Participants

This was a post hoc analysis from an investigator-initiated observational controlled clinical trial, the primary results of which have previously been published (Jacobsen et al., 2021). In brief, the circadian rhythm of plasma magnesium was assessed in ten subjects with CKD and compared to ten control participants as the primary outcome. Plasma magnesium did not appear to have any diurnal variation in either CKD or healthy controls, while urinary excretion of magnesium exhibited a diurnal variation. Furthermore, a circadian rhythm in plasma phosphate, ionized calcium, urinary excretion of phosphate and calcium, plasma PTH, FGF23 and 1,25(OH)_2_ vitamin D was demonstrated, and the diurnal pattern of plasma 1,25(OH)_2_ vitamin D and urinary excretion of calcium was changed in CKD. The study took place at the Department of Nephrology, Herlev and Gentofte Hospital, Denmark.

Eligible participants were ≥18 years of age. The patients with CKD had an estimated glomerular filtration rate (eGFR)(CKD-EPI) between 15 and 30 mL/min/1.73 m^2^ whereas the control group had an eGFR >60 mL/min/1.73 m^2^ and no albuminuria. In the previous six months the participants had plasma ionized calcium, phosphate, and magnesium within the normal range. Patients with diabetes mellitus, plasma PTH >622 pg/mL, previous parathyroidectomy, undergoing treatment with calcimimetics or immunosuppressive drugs, or had active malignancies were excluded.

### 2.2 Study procedures

The participants had blood and urine samples collected every third hour during a 24-hour admission to the hospital. The first morning sample was taken in a non-fasting state, whereas the second morning sample were collected in a fasting state.

During the admission the patients were provided three standard hospital meals and a snack in the afternoon. They were not allowed to sleep or to perform any vigorous exercise during the day, and they slept in the department during the night.

Blood samples collected for analysis of bone turnover markers were placed on ice, centrifuged at 4 °C at 3000 rpm for 10 min and immediately frozen at −80 °C until analysis in one batch. For each assay the sample aliquots were kept frozen until the day of analysis. None of the samples had previously been thawed, and all analyses were performed immediately after thawing the samples.

Serum CTX was measured using the IDS-iSYS CTX (CrossLaps®) assay (Immunodiagnostic Systems, plc, Tyne and Wear, UK). Serum P1NP was measured using the IDS-iSYS intact P1NP assay (Immunodiagnostic Systems). Serum osteocalcin was measured using the N-Mid Osteocalcin assay (Immuodiagnostic Systems). Serum bone-specific alkaline phosphatase (BAP) was measured using the IDS-iSYS Ostase® BAP assay (Immunodiagnostic Systems). Finally, plasma tartrate-resistant acid phosphatase 5b (TRAcP) was measured using the IDS-iSYS BoneTrap® assay (Immunodiagnostic Systems). All assays were carried out on a dedicated automated analyzer, iSYS (Immunodiagnostic Systems) according to the manufacturer's instructions. All assays are chemiluminescence immunoassays. Assay performance was verified using the manufacturers' control specimens. The intermediary precisions expressed as coefficients of variation for CTX were 5.3% (at CTX concentration 213 ng/L), 3.4% (869 ng/L), and 3.5% (2113 ng/L) for iSYS. For P1NP the intermediary precisions were 5.4% (18.96 μg/L), 6.5% (48.48 μg/L), and 6.1% (122.10 μg/L) for iSYS. For osteocalcin the intermediary precisions were 3.0% (8.73 μg/L), 3.6% (27.6 μg/L), and 3.5% (68.7 μg/L). For BAP the intermediary precisions were 8.5% (4.5 μg/L), 7.1% (13.2 μg/L), 3.7% (20.1 μg/L), and 6.3% (52.1 μg/L). Finally, for TRAcP the intermediary precisions were 10.9% (3.2 U/L), 4.8% (6.2 U/L), and 5.4% (9.0 U/L).

### 2.3 Statistics

Data is presented as means and standard deviation or median and interquartile range according to their distribution. They were compared using paired and unpaired *t*-tests or Wilcoxon signed rank test and Mann-Whitney *U* test, as appropriate. Categorical data were presented as numbers and percentages.

CTX, P1NP and osteocalcin were log transformed in order to obtain a normal distribution. The changes within groups and between groups over time points were compared by linear mixed effect models with an autoregressive covariance matrix, which was defined as the primary analysis in the statistical plan. Circadian rhythmicity was also explored by cosinor analysis (Cornelissen, 2014). While the cosinor analysis are searching for a specific circadian pattern, the mixed model are less restrictive and describes changes over time. *P*-values for associations between changes in PTH and bone turnover markers over time were calculated by a generalized estimation equation model to take into account that observations within subjects were not independent. A two-sided *P*-value of <0.05 was considered statistically significant. Statistical analyses were performed in SPSS statistics version 25 and R version 3.6.1.

The study was approved by the Danish Scientific Ethics Committee (H-18037663) and the Danish Data Protection Agency (VD-2018-449).

## 3 Results

The demographics and baseline measurements of the participants are provided in Table 1.

**Table 1:** Demographics.

	CKD (*n* = 10)	Control (n = 10)	Between groups (*P*-value)
Age (years)	71.9 (8.4)	41.4 (10.2)	<0.001
Gender (male)	9 (90%)	5 (50%)	0.141
BMI	27.5 (4.1)	25.5 (3.8)	0.160
eGFR (ml/min/1.73 m^2^)	26 ± 9	105 ± 10	<0.001
Cause of CKD			
Polycystic kidney disease	2 (20%)	–	
Postrenal obstruction	1 (10%)	–	
Infection/rhabdomyolysis	1 (10%)	–	
Glomerulonephritis	1 (10%)	–	
Other	1 (10%)	–	
Unknown	4 (40%)	–	
Smokers	4 (40%)	2 (20%)	NA
Phosphate (mmol/L)	1.09 (0.16)	1.15 (0.13)	0.452
Ionized calcium (mmol/L)	1.18 (0.03)	1.18 (0.02)	0.628
Intact PTH (pg/mL)	40.0 (32.5–50.9)	5.5 (4.7–8.9)	0.024
Fibroblast growth factor 23 (pg/mL)	142.0 (49.5–197.8)	9.4 (7.4–13.6)	0.005
1,25 (OH)_2_ vitamin D (pmol/L)	67.0 (59.0–87.0)	104.5 (72.3–121.0)	0.019
25 OH vitamin D (nmol/L)	87 (24)	62 (18)	0.018

All participants were Caucasian, none of them had experienced a bone fracture during the last 12 months, and none had known liver disease. In the CKD group only one woman was included, and she was postmenopausal. In the control group five women were included and one was postmenopausal.

**Fig. 1:**
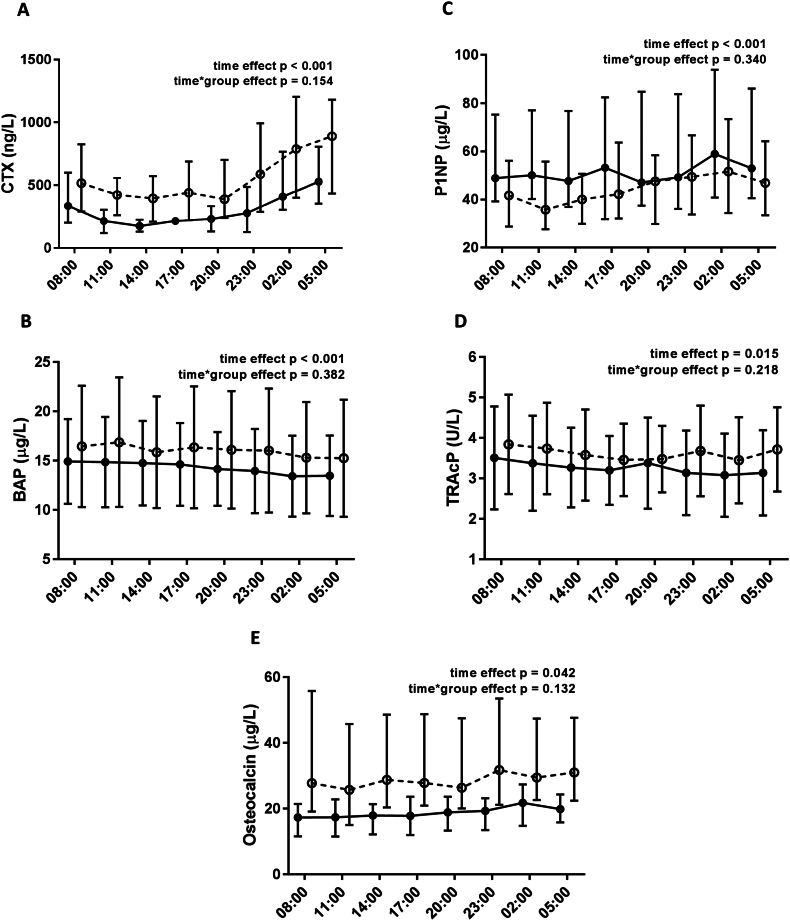
Circadian rhythm of bone turnover markers in patients with CKD and healthy controls. Circadian rhythm was analysed by mixed effect model. The time effect determined the rhythmicity in the parameter whereas the time*group interaction was a determination of differences in the circadian rhythm between groups. A: CTX, B: BAP, C: P1NP D: TRAcP, and E: Osteocalcin CTX and osteocalcin are presented as median (interquartile range) BAP, P1NP and TRAcP are presented as mean ± standard deviation CTX = C-terminal telopeptide of type I collagen; BAP = bone-specific alkaline phosphatase; P1NP = N-terminal propeptide of type I procollagen; TRAcP = Tartrate-resistant acid phosphatase; CKD = chronic kidney disease.

**Fig. 2:**
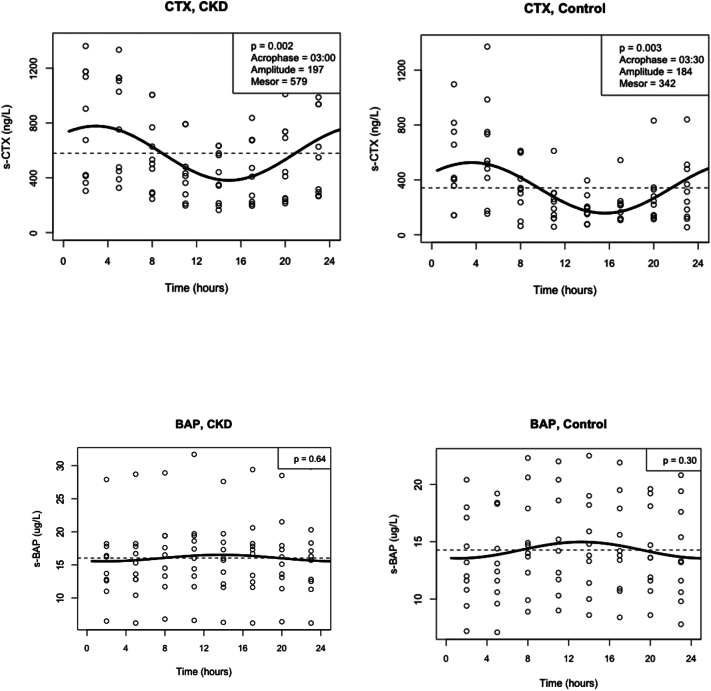

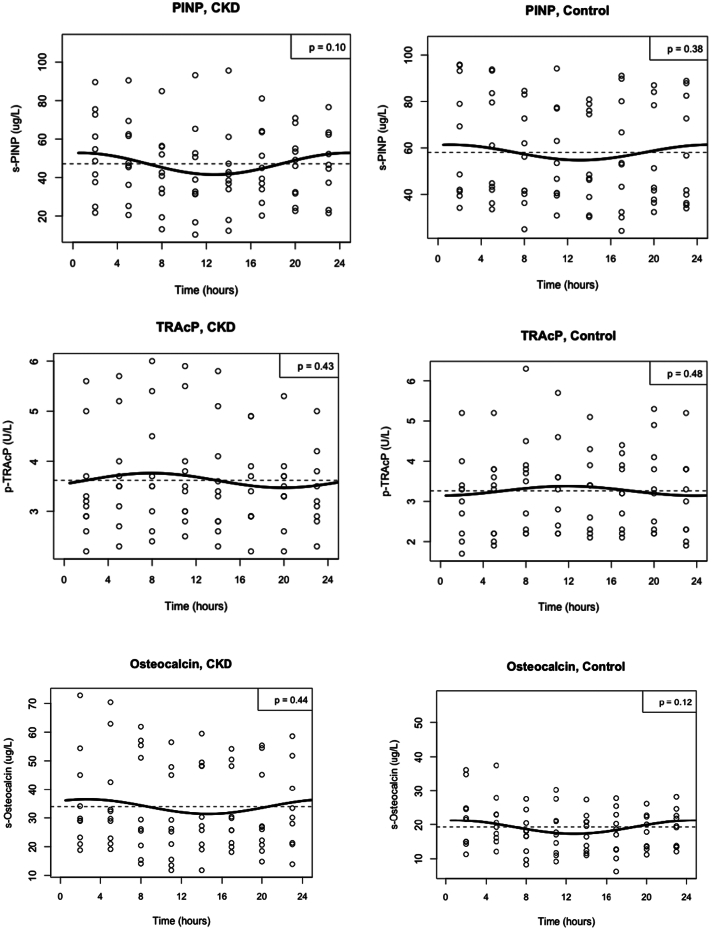
Cosinor analyses of CTX, BAP, P1NP, TRAcP, and osteocalcin in patients with CKD and healthy controls. In CTX a significant circadian rhythm was found in both groups by cosinor analysis with an acrophase around 3:00 AM. No circadian rhythm was found for any other of the bone turnover markers by cosinor analyses. CTX = C-terminal telopeptide of type I collagen; BAP = bone-specific alkaline phosphatase; P1NP = N-terminal propeptide of type I procollagen; TRAcP = Tartrate-resistant acid phosphatase.

We examined the circadian variation of the various markers of bone turnover by a linear mixed effects model and observed a significant effect of time on all bone turnover markers, thus demonstrating a circadian variation in CTX (*p* < 0.001), P1NP (p < 0.001), osteocalcin (*p* = 0.042), BAP (p < 0.001) and TRAcP (*p* = 0.015) (Fig. 1). This was confirmed for CTX by cosinor analyses in both CKD (*p* = 0.003) and controls (*p* = 0.002) with an acrophase at around 3 o'clock a.m. (Fig. 2), however, we were unable to find significant rhythmicity for P1NP, osteocalcin, BAP or TRAcP by cosinor analysis. We found no interaction between time and group in any of the bone markers and thus no evidence that CKD affects their circadian rhythm compared to healthy controls (CTX (*p* = 0.154), P1NP (*p* = 0.340), osteocalcin (*p* = 0.132), BAP (*p* = 0.382) and TRAcP (*p* = 0.218)) (Fig. 1A–E). After adjustment for age and gender, there were still no significant interaction between time and group in any of the parameters (CTX (p = 0.154), P1NP (p = 0.340), osteocalcin (*p* = 0.134), BAP (p = 0.382) and TRAcP (p = 0.218)). Overall, both CTX (*p* = 0.025) and osteocalcin (*p* = 0.007) were significantly higher in CKD compared to controls, while there was no overall difference between CKD and control for P1NP (*p* = 0.273), BAP (*p* = 0.449) or TRAcP (*p* = 0.438). The level of CTX (*p* = 0.005) and osteocalcin (*p* = 0.000) were significantly influenced by age, whereas the levels of the other parameters were not (*p* > 0.05). However, adjustment for age and gender did not influence the overall difference with higher levels of CTX and osteocalcin in patients with CKD compared to healthy controls, and no difference between groups in the overall level of the other bone turnover markers (data not shown). There was a positive correlation between changes in PTH and changes in bone turnover markers although this was not significant for any of the parameters: CTX (*r* = 0.24, *p* = 0.091), P1NP (*r* = 0.11, *p* = 0.14), BAP (*r* = 0.08, *p* = 0.39), TRAcP (*r* = 0.05, p = 0.14), osteocalcin (*r* = 0.18, *p* = 0.089).

The influence of fasting was examined by comparing the levels of bone turnover markers in a non-fasting state at 8 a.m. compared to levels in a fasting state 8 a.m. the following day. CTX was significantly higher in the fasting state in both the CKD (39%) (*p* = 0.005) and in the control group (33%) (*p* = 0.016). Fasting did not influence the other bone turnover markers in the CKD group, while in the control group P1NP and osteocalcin were 10% (*p* < 0.001) and 21% (*p* = 0.007) higher, respectively, and BAP was lower (6%) (p < 0.001) in the fasting state (Table 2).

**Table 2:** Bone turnover markers in non-fasting and fasting state.

	CKD		Control	
	Non-fasting	Fasting	Non-fasting	Fasting
CTX (ng/L)	517 (290–827)	828 (434–1216)⁎	336 (201–600)	472 (306–715)⁎
PINP (μg/L)	41.7 (28.8–56.1)	46.3 (30.9–66.9)	48.9 (39.2–75.3)	53.6 (42.2–82.8)⁎
Osteocalcin (μg/L)	27.75 (19.2–55.8)	29.85 (22.4–46.8)⁎⁎	17.30 (11.6–21.4)	20.95 (15.0–29.8)⁎
Bone-specific alkaline phosphatase (μg/L)	16.5 (6.2)	15.5 (6.6)	14.9 (4.3)	14.0 (4.2)⁎
TRAcP (U/L)	3.84 (1.23)	3.89 (1.25)	3.51 (1.27)	3.59 (0.99)

All values are mean and standard deviation except for CTX and osteocalcin which are given as median and interquartile range.

⁎ P < 0.05: fasting versus non fasting state (paired *t*-test or Wilcoxon signed ranks test).

⁎⁎ P < 0.05: CKD versus Control in fasting state (unpaired t-test or Mann-Whitney *U* Test).

## 4 Discussion

A circadian rhythm in bone turnover markers is well known in healthy individuals (Diemar et al., 2022).In the present study, we demonstrate for the first time that a circadian rhythm in the bone turnover markers is also present in CKD, although this does not appear to be caused by CKD in itself.

When bone collagen is broken down, the cleavage of cross-linked type 1 collagen releases CTX into the blood and plasma CTX is therefore considered a marker of bone resorption (Chubb, 2012). In several studies of individuals with normal kidney function a pronounced circadian rhythm of CTX has been demonstrated similar to the findings in the present study (Redmond et al., 2016; van der Spoel et al., 2019; Bjarnason et al., 2002; Swanson et al., 2017). The circadian rhythm of CTX has been shown to be unaffected by gender, age, postmenopausal status, five days of bedrest, or serum levels of cortisol (Qvist et al., 2002). Fasting has been shown to reduce the circadian variation of CTX, and oral or intravenous glucose induces an acute decrease in CTX. Thus, the circadian rhythm of CTX may at least partly be explained by food intake. The mechanism of reduction in CTX seems related to an increase in the incretin hormones, especially gastric inhibitory peptide (GIP) (Westberg-Rasmussen et al., 2017). Indeed, expression of GIP receptors have been demonstrated on osteoclasts, and GIP leads to inhibition of osteoclast activity (Zhong et al., 2007). We did not examine the circadian rhythm in a fasting state, but we found a significantly higher level of CTX in the fasting state in both groups and therefore the circadian variation in CKD is likely also influenced by food intake.

The patients with CKD presented with the same pattern of diurnal variation in CTX as the control group with a maximum at night around 3 a.m. and nadir in the afternoon. As such, the present findings do not support a disturbance in the circadian pattern of bone resorption to be the cause of bone disease in CKD.

CTX is associated with increased fracture risk and recommended for monitoring of treatment response (Johansson et al., 2014; Morris et al., 2017). However, the usefulness of CTX as a marker of bone resorption in patients with eGFR <30 mL/min/1.73 m^2^ is limited, since CTX undergoes renal excretion and as such the overall higher level of CTX in the CKD group was to be expected. Reference ranges for different levels of reduced kidney function have not been established and individual changes in CTX may occur as kidney function declines, which makes CTX inappropriate for monitoring of treatment response in patients with CKD. Additionally, the applicability of CTX as a predictor of bone turnover has been examined by comparing bone turnover markers with bone histomorphometry in patients with CKD and the ability of CTX to predict bone turnover was insufficient (Salam et al., 2018).

TRAcP is expressed by bone resorbing osteoclasts and, like CTX, TRAcP is a marker of bone resorption (Halleen et al., 2006). The circadian rhythmicity of TRAcP has not been previously described in human studies, but in rats and calves a circadian rhythm of TRAcP has been demonstrated (Shao et al., 2003; Matsuo et al., 2014). The present study confirms a circadian rhythm of TRAcP in both the CKD and healthy controls. No differences in the overall level of TRAcP was found between the two groups, which is in accordance with TRAcP being unaffected by the kidney function. Changes in TRAcP may therefore be used to reflect changes in bone resorption in patients with CKD (Yamada et al., 2008).

Several studies have demonstrated a circadian rhythmicity in the bone formation markers P1NP, osteocalcin and BAP (Greenspan et al., 1997; Redmond et al., 2016; van der Spoel et al., 2019; Hygum et al., 2019; Schlemmer et al., 1997), although the definition of circadian rhythm and the statistical approach differed markedly between the studies. In the present study, we found a circadian pattern in the bone formation markers in both groups when examined with linear mixed effects models. However, the cosinor analysis could not detect any circadian rhythmicity in the individual groups, probably due to the small number of participants in the individual groups and the rhythmicity in the bone formation markers being less pronounced than for CTX. This is in accordance with the study by Schilperoort et al. (2020), which found a more pronounced rhythmic expression of circadian clock genes in osteoclasts, suggesting that bone resorption exhibits the predominant circadian rhythm in bone tissue.

Osteocalcin is excreted by the kidneys and we observed a higher level of osteocalcin in the CKD group compared to the control group (Silaghi et al., 2019). Accordingly, the higher levels of CTX and osteocalcin in the CKD group were likely due to the reduced kidney function and not a reflection of an elevated bone turnover. Also, although PTH levels were higher in the CKD group, the other bone turnover markers were at similar levels in both groups.

Hyperparathyroidism is common in CKD and although PTH stimulates bone resorption we did not find a statistically significant correlation between PTH and bone turnover markers. This may be due to other factors than PTH influencing bone turnover. Furthermore, both the skeletal resistance to PTH in CKD and analytical aspects (determination of both active and inactive fragments of PTH) (Evenepoel et al., 2016) may have affected the chance of detecting a significant association.

In the CKD group, the influence of fasting did not affect any of the bone turnover markers except for CTX. In the control group, a significant influence of fasting was present for P1NP, osteocalcin, and BAP. However, the differences were minor and fasting is not usually recommended before measurement of these bone formation markers. The clinical significance of these findings are therefore likely to be minimal.

The present study has some limitations. A larger number of participants may have uncovered small differences between groups or in the circadian rhythm. Also, differences in age, gender and postmenopausal status between the groups as well as further standardization of the examination conditions may have influenced the results. Blood samples were drawn every third hour and an increased frequency of blood sampling may have provided more detailed curves for the circadian rhythm.

## 5 Conclusion

A similar circadian rhythm in bone turnover markers is present in both patients with CKD and healthy controls, although the clinical implications of this remains to be determined. The present findings do not support a disturbance in the circadian rhythm of bone turnover to be the cause of bone disease in CKD.

## CRediT authorship contribution statement

Hansen D: conceptualization, formal analysis, resources, writing original draft, project administration. Bressendorff I: conceptualization, investigation, writing review and editing, visualization. Nordholm A: formal analysis, writing-review and editing, visualization. Møller AS investigation. Klausen TW: methodology, formal analysis. Jørgensen NR: investigation, writing-review and editing, funding acquisition. All authors approved the final paper.
